# Observation of the linewidth broadening of single spins in diamond nanoparticles in aqueous fluid and its relation to the rotational Brownian motion

**DOI:** 10.1038/s41598-018-33041-6

**Published:** 2018-10-03

**Authors:** Masazumi Fujiwara, Yutaka Shikano, Ryuta Tsukahara, Shinichi Shikata, Hideki Hashimoto

**Affiliations:** 1 Department of Chemistry, Osaka City University, Sumiyoshi-ku, Osaka, 558-8585 Japan; 2 School of Science and Technology, Kwansei Gakuin University, Sanda, Hyogo 669-1337 Japan; 3 Institute for Molecular Science, National Institutes of Natural Sciences, Okazaki, Aichi 444-8585 Japan; 4 Institute for Quantum Studies, Chapman University, Orange, California 92866 USA; 5 Research Center for Advanced Science and Technology (RCAST), The University of Tokyo, Meguro-ku, Tokyo, 153-8904 Japan; 6 Quantum Computing Center, Keio University, Hiyoshi, Yokohama 223-8522 Japan

## Abstract

Capturing the fast rotational motion of single nanoparticles has been hindered owing to the difficulty of acquiring directional information under the optical diffraction limit. Here, we report the linewidth broadening of the electron spin resonance of single nitrogen vacancy (NV) centers that matches the rotational diffusion constant of the host nanodiamonds. When nanodiamonds are gradually detached from the substrates that they were fixed to, their optically detected spin resonance peaks are broadened by 1.8 MHz, which corresponds to the rotational diffusion constant of nanoparticles with a diameter of 11.4 nm from the Einstein–Smoluchowski relation.

## Introduction

Rotations of objects characterize one of the fundamental properties of the dynamics and can be measured by various optical techniques. It is however significantly limited on the nanoscale owing to the difficulty of acquiring directional information under the optical diffraction limit. For characteristic dimensions above the sub-micron scale, optical microscopy provides ways to observe the rotational motion of small particles, such as capturing the anisotropic morphology of particles^1–3^, polarization-sensitive optical detection of metallic nanorods^4,5^, and extracting rotational information based on the detailed analysis of the 3D-translation dynamics^6^. On the other hand, at the atomic scale, fluorescence depolarization spectroscopy provides information on the rotational Brownian motion (diffusion) of ensembles of molecules^7^. However, detecting rotational motion on the nanoscale, which lies between the atomic and the micron scales, has not been hitherto well explored.

The difficulty of detecting rotational motion on the nanoscale arises from two major technical challenges. First, it is difficult to capture the shapes of nanoparticles by optical means due to their sizes being smaller than the optical diffraction limit. Emission (either fluorescence or scattering) from nanoparticles is treated as coming from a point light source and cannot provide the directional information. Second, the timescale of the rotational diffusion of nanoparticles is quite fast with a high dynamic range; for example, in water, it can vary from millisecond to microsecond for particle diameters from 100 nm to 10 nm, which is several orders of magnitude faster than that of micron-scale particles (1 μm gives 1.45 Hz)^8^. Most of the image-based optical techniques cannot provide such high-frequency detection. Thus, the detection of the rotational motion of nanoparticles has been elusive.

A new approach that could access the rotational motion of single nanoparticles is to exploit the electron spins of nitrogen vacancy (NV) centers in nanodiamonds. Nanodiamonds can be used as very stable fluorescence nano-light emitters when incorporating NV centers^9–11^. The intensity of the NV fluorescence is electron-spin-dependent and can be affected by the nanoscale local environment of the nanodiamonds, such as magnetic fields, electric fields, and temperature^12,13^, which allows for quantum-enhanced nanoscale sensing^14–16^. Applications of NV centers now extend to 3D-orientation tracking of nanoparticles^17–20^ and nanoscale thermometry in living cells^21,22^. The electron spins of NV centers are able to sense the rotational motion of the host nanodiamonds by exploiting the orientation of the spin systems. The slow movement of nanodiamonds has been successfully detected in living cells within hours by measuring the Zeeman shift of the spin resonance peak under a static external magnetic field^20^. The faster timescale of nanoparticle rotation in the order of the rotational diffusion rate (MHz) has also been theoretically predicted^23^ as the random walk of the spin precession angle is accumulated as the the geometric phase fluctuation of the NV quantum system; the geometric phase fluctuation leads to dephasing of the electron spin coherence and thus broadens the electron spin resonance line of NV centers in cw-ODMR detection (continuous wave optically detected magnetic resonance)^23–26^. However, this theoretical prediction has not been confirmed yet.

Here, we report the linewidth broadening of the ODMR lines of single NV centers that matches the rotational diffusion constant of the host nanodiamonds. Single nanodiamonds incorporating single NV centers are slowly detached from the host substrates in an aqueous buffer solution. Continuous optical measurement of the NV centers during the detachment process allows for measuring the ODMR spectra of the same NV centers when the nanodiamonds are either fixed to the substrate or fluctuating during the detachment. The ODMR line is clearly broadened by 1.8 MHz (full width at half maximum, FWHM) by the nanodiamond fluctuation. The observed broadening shows good agreement with the diffusion constant of nanodiamonds with a diameter of 11.4 nm, which is derived from the Einstein–Smoluchowski relation. Our findings may provide a way to measure the rotational Brownian motion of single nanoparticles and enable the exploration of nano-scale fluid mechanics.

## Results

### Confocal fluorescence microscopy of singe NV centers in nanodiamonds in aqueous buffer solutions

**Figure 1:**
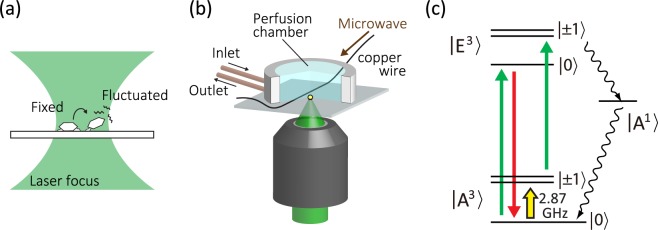
(**a**) Schematic drawing of a single nanodiamond gradually detached from a coverslip it was attached to. The nanodiamond orientation (NV axis) is fluctuating to detach. (**b**) Close up of the central part of the experimental setup. The nanodiamond is placed in a home-made perfusion chamber that simultaneously allows exchange of the solution and the optical experiment. A thin copper wire is fed through into the chamber for the spin excitation. Single NV centers hosted in nanodiamonds are excited by 532-nm laser light and are observed with red-shifted fluorescence collected through the same microscope objective. (**c**) Energy diagram of NV centers. The main optical transition occurs between the ground state (|*A*^3^〉) and the excited state (|*E*^3^〉). Microwave excites the electron spin from |0〉 to |±1〉 in the sub-states of |*A*^3^〉, followed by spin-conserving optical transitions and intersystem crossing from |±1〉 states in |*E*^3^〉 to the lower singlet state, (|*A*^1^〉). The population in |*A*^1^〉 is nonradiatively relaxed to the spin |0〉 state of the triplet ground state, which causes a decrease of the fluorescence intensity.

**Figure 2:**
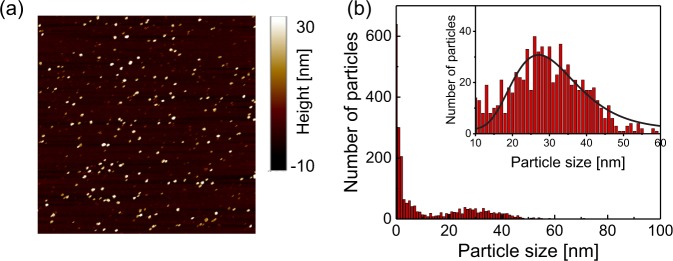
(**a**) AFM topography image of the nanodiamonds deposited on the coverslip. The area is 8 × 8 μm^2^. (**b**) Corresponding particle size histogram. The solid line is a log-normal curve fitting to the data. Note that there are number of particles with sizes smaller than 10 nm. These particles are considered to be debris included in the original suspension.

Figure 1(a) shows a schematic drawing, which depicts nanodiamonds that are about to be detached from the coverslip. We use commercially available nanodiamonds with a median size of 25 nm (Microdiamant, MSY 0–0.05). A droplet of the nanodiamond suspension is spin-coated on a coverslip. The uniform nanodiamond distribution on the coverslip is confirmed by atomic force microscopy as shown in Fig. 2. We then fabricate a home-made perfusion chamber on the coverslip, which simultaneously allows liquid exchange and optical observation as shown in Fig. 1(b) (see Methods and Supplementary Information for the details). Distilled water is sent into the perfusion chamber to immerse nanodiamonds in liquid, followed by subsequent optical and ODMR measurements. The total volume of the tube line and the perfusion chamber is 490 μL. The liquid flow keeps running with the flow rate of 80 μL · min^−1^ in the subsequent experiments unless specifically mentioned.

**Figure 3:**
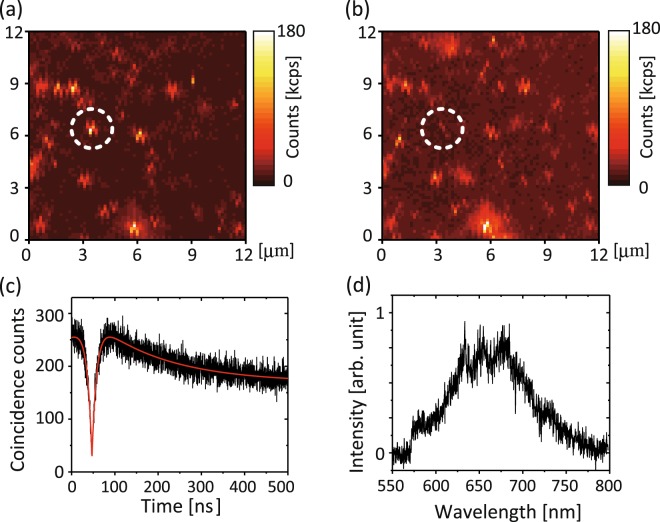
(**a**) Confocal fluorescence scanning image of nanodiamonds deposited on a coverslip and immersed in water. The fluorescent nanodiamond indicated by the dashed circle is to be detached. (**b**) Scanning image of the same region after the nanodiamond is detached. (**c**) The second-order photon correlation histogram and (**d**) fluorescence spectrum of the nanodiamond, when it is fixed on the coverslip in water. The distortion of the fluorescence spots is due to the hysteresis of the piezo scanner.

Figure 3(a) shows a confocal fluorescence scanning image of nanodiamonds fixed on a coverslip under the flow of distilled water. There are isolated nanodiamonds showing fluorescence signals, most of which are ascribed to NV centers. Figure 3(c,d) show the second-order photon correlation histogram and fluorescence spectrum of the nanodiamond indicated by the dashed circle, respectively. The photon-correlation histogram shows an antibunching dip with *g*^(2)^(0) = 0.16 (the time origin *t*_0_ = 47 ns) and a bunching shoulder at *t* ~ 100 ns due to the population exchange with the nearby metastable state^27^ (see Fig. 1(c)), which clearly indicates incorporation of a single negatively charged NV^−^ center. The temporal profile of the photon correlation data can be fitted with an equation reported elsewhere^27^, which yields *τ*_1_ = 11 ns and *τ*_2_ = 166 ns. The fluorescence spectrum is another clear signature of the presence of NV^−^ centers; the zero-phonon line is observed at 634 nm, accompanied by a broad phonon sideband up to 750 nm^28,29^.

We then replace the water with a buffer solution of pH = 7.5 and measure the ODMR spectra. The pH of the solution is changed stepwise (7.5 → 8.2 → 9.1 → 9.9) by adding a carbonate buffer solution. After pH = 9.9 is reached, it is brought back to pH = 9.1 by adding HCl. A single process of changing ΔpH~1 takes about an hour that includes adjusting the pH of the reservoir, liquid circulation, and ODMR characterization. The total time to change from water to pH = 9.1 (the final pH in this experiment) is about 6 hours. When the pH is changed back to pH = 9.1, the fluorescence from the nanodiamond starts to fluctuate, as the nanodiamond is about to detach from the coverslip. During the time of the nanodiamond fluctuation of several minutes, we are able to measure the ODMR spectrum, which will be described in the next section. The nanodiamond finally moves away due to the continuous flow in the perfusion chamber.

Figure 3(b) shows a confocal scanning image of the same region as imaged in Fig. 3(a) after the nanodiamond is completely detached. There is no more prominent fluorescent spot remaining inside the dashed circle, while other fluorescent nanodiamonds are still located at the same positions. There remain residues that show very small fluorescence of 32 kcps; this is about 5 times smaller than the average fluorescence count of single NV centers detected in water in our laboratory setup (~150 kcps). We therefore confirm that only the nanodiamond indicated by the dashed circle is removed during the pH change. Note that there are some new fluorescent blurred spots emerged in Fig. 3(b) (see Supplementary Fig. S1 for the details). These spots were created by the green laser excitation when we stopped the liquid flow during the experiment. Without the continuous liquid flow, the green laser excitation gradually generates such fluorescent spots (the fluorescence eventually grows much brighter than the single NV fluorescence if the liquid flow is stopped for a long time). This is probably because nanodiamonds detached from other locations (beyond the imaging region) are accumulated around the laser spot due to the strong green laser excitation (optical forces, laser heating, etc.^30^). This phenomenon is more prominent in more acidic pH buffer solutions, which may be related to the zeta potential of nanodiamonds, as nanodiamonds show lower negative zeta-potentials for more acidic pH^31,32^.

### ODMR measurements on single NV centers

**Figure 4:**
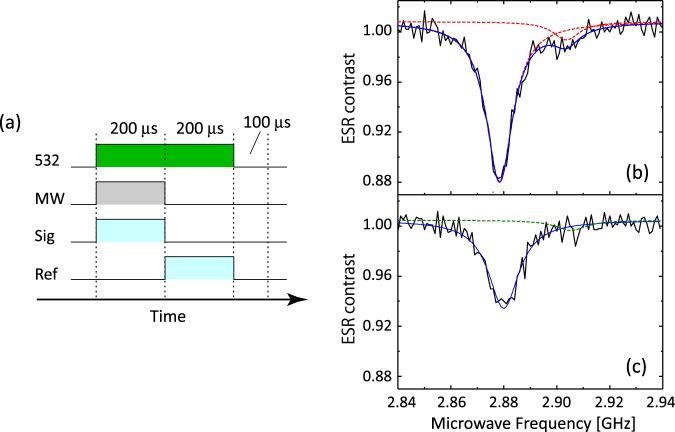
(**a**) Schematic illustration of the gated photon counting for the ODMR measurements. The APD detection is gated for microwave irradiation ON and OFF. The APD gate width is 200 μs common to the both gates, giving ${\rm{\Delta }}{I}_{{\rm{PL}}}={I}_{{\rm{PL}}}^{{\rm{ON}}}/{I}_{{\rm{PL}}}^{{\rm{OFF}}}$. The repetition rate of the gating (including laser off time of 100 μs) is 2 kHz. The 532-nm green laser irradiation is continuously ON during these gating periods. 532: 532-nm green laser pulse. MW: microwave pulse. Sig: photon counting while the microwave is ON. Ref: photon counting while the microwave is OFF. (**b**) ODMR spectra of the nanodiamond indicated by the dashed circle (Fig. 3) when it is fixed on the coverslip and (**c**) is about to detach. The blue solid lines in (**b** and **c**) are the Lorentzian fits to the data, and the red dashed lines in (**b**) are the 2-peak components of the fitting. The olive dashed line in (**c**) is the reproduced curve for the minor peak, which is almost buried in the noise, calculated by taking account of the reduction of the main peak contrast (or area).

We measure ODMR spectra of the nanodiamonds throughout the course of the pH change. Figure 4(a) shows a pulse sequence used to obtain the ODMR spectra of single NV centers in nanodiamonds. The microwave excitation and APD detection are gated with a common gate width of 200 μs and a repetition rate of 2 kHz, in order to extract spin-dependent signals out of the fluorescence fluctuation due to the environmental noise such as defocusing of the laser spot, heating by the microwave irradiation, and the nanodiamond fluctuation (see Methods)^19^. The fluorescence intensities with/without the microwave excitation (${I}_{{\rm{PL}}}^{{\rm{ON}}}/{I}_{{\rm{PL}}}^{{\rm{OFF}}}$) are measured, and their ratio ${\rm{\Delta }}{I}_{{\rm{PL}}}={I}_{{\rm{PL}}}^{{\rm{ON}}}/{I}_{{\rm{PL}}}^{{\rm{OFF}}}$ (ODMR contrast) is plotted as a function of the microwave frequency. The external magnetic field is not applied in this experiment.

Figure 4(b) shows an ODMR spectrum of a single NV center when the nanodiamond is fixed to the coverslip in distilled water (Fig. 3(a)). The ODMR spectrum is composed of two Lorentzian peaks. Curve fitting with a two-peak Lorentzian profile determines that the major fluorescence peak is located at 2.8784(1) GHz with a linewidth of 12.2(4) MHz (FWHM), and another associated minor peak is located at 2.9036(11) GHz with a linewidth of 12.1(40) MHz (FWHM). These two peaks are the result of intrinsic splitting of the magnetic sublevels of |±1〉 spin states by lattice strain in the nanodiamond, which is often observed in nanodiamond NV centers^33^. We note that the presented errors are from the curve fitting.

Figure 4(c) shows the ODMR spectrum of the single NV center while the fluorescence from the nanodiamond is fluctuating in the final buffer solution at pH = 9.1. The major peak is clearly broadened, and the other minor peak is weakened. We fit the data with a single Lorentzian profile, since the intensity of the minor associated peak is comparable to the noise level, which makes curve fitting difficult (the peak still exists at around 2.905 GHz). The major peak is located at 2.8800(3) GHz with a FWHM linewidth of 14.0(9) MHz. The linewidth is broadened by 1.8(9) MHz compared with that of the fixed configuration (Fig. 4(b)). We note that the contrast of the major peak is decreased by 45% (35% in peak area) compared to that shown in Fig. 4(b). This decrease buries the minor peak under the noise, which justifies the use of a single Lorentzian profile for the curve fitting (we discuss this issue in Discussion).

## Discussion

**Figure 5:**
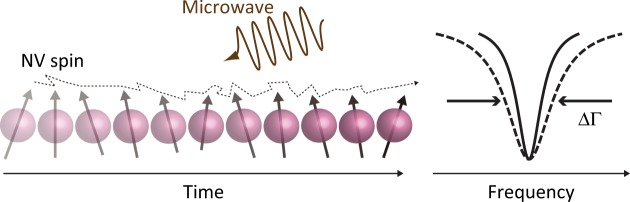
A shcematic illustration of the phase accumulation of the nanodiamond-NV spin systems during the rotational Brownian motion as a possible mechanism of the ODMR linewidth broadening.

In this paper, we reported the linewidth broadening of the ODMR peaks of single NV centers that occur during the detachment from the coverslip. Surprisingly, the observed linewidth broadening matches the rotational diffusion constant of the host nanodiamonds. Fast rotation of nanodiamonds adds a geometric phase to the time evolution of the NV spin system as theoretically studied in refs^23,24^. When the spin measurement time is sufficiently long compared with the rotational diffusion constant of the nanodiamonds, the final ODMR signal is averaged over the entire ensemble of initial orientations and rotational trajectories. The random fluctuation of the geometric phase creates an additional decay channel for the quantum superposition, which modifies the spin coherence time as ${T}_{2}^{\ast }\to {T}_{2}^{\ast }+{k}_{d}^{-1}$ (see Fig. 5). Here, *k*_*d*_ is the rotational diffusion constant of nanodiamonds and, according to the Einstein–Smoluchowski relation, is given by

1 $${k}_{d}(\,=\frac{{\rm{\Delta }}{\rm{\Gamma }}}{2})=\frac{{k}_{B}T}{8\pi {(d\mathrm{/2)}}^{3}\eta },$$

where ΔΓ, *k*_*B*_, *T*, *d*, and *η* are the observed linewidth broadening in the ODMR spectrum, Boltzmann constant, temperature, diameter of the nanoparticles, and viscosity of the surrounding medium, respectively. Note that ΔΓ = 2*k*_*d*_, considering the time–frequency transformation relation of the exponential decay and the Lorentzian line shape.

In the present case, the surrounding medium is a carbonate buffer-based solution that has a viscosity of *η* = 0.97 mPa at room temperature (T = 293 K) determined by a viscotester (Toki Sangyo, RE100L). The spin measurement time is 200 μs, sufficiently longer than the rotational diffusion time of the nanodiamonds, as it corresponds to the diffusion constant of nanoparticles with a diameter of 66 nm (5 kHz). The observed linewidth broadening is hence calculated to be the rotational diffusion constant of nanoparticles with a diameter of *d* = 11.4(2) nm. The size statistics of our nanodiamonds exhibit a mean diameter of 30 nm with a distribution ranging from 10 to 50 nm on the basis of the AFM topography image shown in Fig. 2. The observed linewidth is within the range of the particle distribution, thus indicating that it comes from the rotational Brownian motion.

While the results indicate the effect of the rotational Brownian motion on the ODMR peaks of NV centers, there still remain questions that need to be addressed to fully understand the presented results. First, laser heating of the nanodiamonds by the 532-nm green laser may affect the linewidth broadening as it can change the local viscosity. The local temperature in the present situation can rise up to, for example, 90 °C (slightly lower than the boiling point of water), which reduces the viscosity of water to almost one third of the original value^34^ (1.0 mPa at 20 °C → 0.31 mPa at 90 °C). The change of the local viscosity may lead to overestimation of the linewidth broadening by a factor of 3, giving the corresponding particle size of *d* = 16.4(3) nm. Such temperature change, however, might not occur in the present situation, since we did not observe the peak shift that corresponds to this temperature change of −0.7 MHz (the temperature change causes the peak shift of −74 kHz · K^−1^)^35^.

Interestingly, the linewidth broadening of the ODMR peaks has been observed in optically trapped nanodiamonds with a relatively large diameter of 74 nm^17^. The nanodiamonds used in ref.^17^ have a mean particle size of 74 nm based on the dynamic light scattering data and are trapped in a viscous solution (glycerin: water = 5:1, viscosity 132 mPa at 20 °C)^36^, which gives *k*_*D*_ of ~24 Hz. A detectable broadening was confirmed by statistically comparing the ODMR spectra of the trapped nanodiamonds with those of the fixed ones (Fig. S9 in ref.^17^) and qualitatively ascribed to the precession of the NV axis. Further quantitative evaluation would be important to reveal the detailed mechanism of the linewidth broadening.

Second, the contrast (peak area) of the major peak in Fig. 4(c) is decreased by 45 (36.5) % compared to that in Fig. 4(b). This peak reduction results in the associated minor peak being buried under the noise level, which makes it impossible to fit with the original double Lorentzian peak profile. By applying the same change (45%-contrast (36.5%-peak-area) reduction and 1.6-MHz-frequency shift) to the fitting parameters of the minor peak in Fig. 4(b) and keeping other parameters fixed, we reproduce the simulated curve for the minor peak as shown in Fig. 4(c) (dashed olive line). Table S1 (Supporting Information) summarizes the fitting parameters of the minor associated Lorentzian peak in Fig. 4(b) and the parameters to reproduce the minor peak in Fig. 4(c). The peak contrast 2*A*/*πw* (or area *A*) is reduced by 45 (36.5) % after the detachment, and the peak position (*ω*_1_) is shifted by 1.6 MHz. We apply these percentage peak reductions and frequency shifts to the minor peak while using the common *y*_0_ for the major peak. The reproduced minor peak is smaller than the the noise level (±0.01), which justifies the use of a single Lorentzian profile for curve fitting.

**Figure 6:**
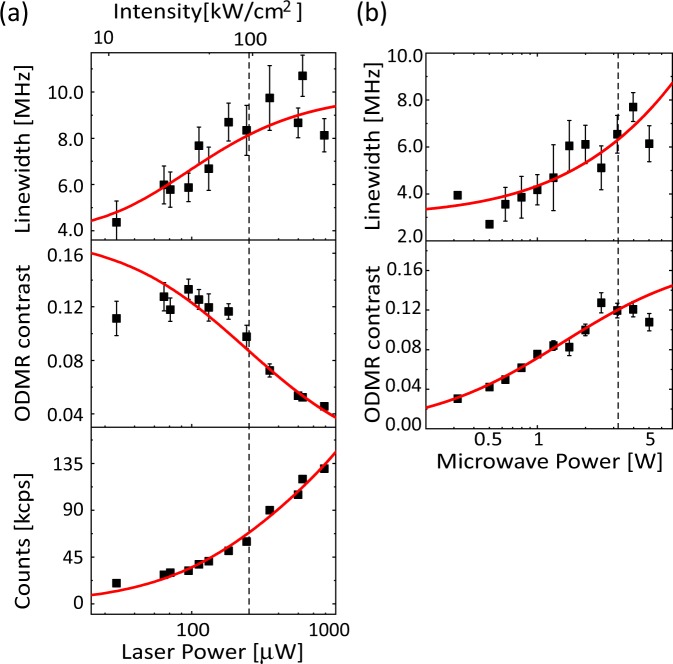
(**a**) Laser power dependence of the peak parameters (linewidth and contrast). The dependence of the photon counts is also shown in the bottom panel. The red solid lines are the theoretical fit to the data based on the quantum dynamics of the NV electronic systems^10,37,50^. The saturation intensity is 0.34 mW based on the fitting in the bottom panel. (**b**) Microwave power dependence of the peak parameters with the theoretical fits^37^. The microwave power is an input power to the antenna in the impedance mismatching condition. The error bars are fitting errors (1*σ*). The dashed lines indicate the power used for the measurement of Fig. 4. Note that the photon count in this figure is smaller than the value shown in Fig. 3(a) (150 kcps). This difference comes from the slight difference of the optical alignment tuning; it reproduces the same condition as used in Figs 3 and 4.

Both the microwave power and the laser excitation power can affect the peak parameters (linewidth, contrast), as the ODMR linewidth exhibits corresponding dependences^37^. We measured the dependence of the ODMR peaks on both the microwave power and laser power for nanodiamonds fixed to a coverslip in water to estimate the effect of these parameters on the reduction of the peak area (and linewidth broadening). Figure 6(a,b) show the dependences of the ODMR spectral line parameters (linewidth and contrast) on the microwave power and laser power, respectively. For the laser power, the dependence of the photon counts is also shown. In the experiments of Fig. 4, the laser power was 250 μW, and the microwave power was 3.2 W (35 dBm), as indicated by the dashed lines in the figures. From the data, we can estimate the uncertainty through the fluctuations of the laser power and microwave power. The linewidth can be broadened by ±0.2 MHz by the typical microwave power uncertainty of 1% and ±0.2 MHz by the typical laser intensity fluctuation of 5–10%. The uncertainty of the peak reduction are 0.1% and 5–10%, respectively. The observed linewidth broadening and the peak reduction therefore do not originate from the power change of the microwave and the laser, confirming the origin of the linewidth broadening as the rotation Brownian motion of the nanodiamonds.

Third, the surface charge of the nanodiamonds (or adsorption of ions) may affect the ODMR spectral profiles due to their small particle size; it is known that small nanodiamonds are more likely to have an inhomogeneous surface and their instability is caused by surface chemistry, which may be prominent in the present experiment performed under the ionic buffer solution. However, we have not observed such a drastic change in linewidth for the other nanodiamonds which were not detached during the long-term and pH-change experiments^38^. The variation of the linewidth over the pH change from 4 to 11 remained less than ±0.3 MHz and the 24-hour long-term variation less than ±0.3 MHz.

Fourth, the high concentration of ions in the buffer solution does not affect the ODMR spectra significantly. We have observed the ODMR spectra of single NV centers in nanodiamonds immobilized on the coverslip while changing the liquid fluid from the distilled water to pH-8.8 buffer solution and have not detected the significant change of the linewidth (see Fig. S4). The observed linewidth changes of the data are 0.71 MHz for the left peak and 0.49 MHz for the right peak, which are significantly smaller than the linewidth change of 1.8 MHz of Fig. 4c. Note that the resonance peak positions are changed by 4 MHz (left peak) and 1 MHz (right peak). We have determined that the uncertainty of the positional determination is 1–3 MHz both in water and the buffer solution and therefore the observed peak shift is not significant.

Considering these possible origins and discussions, the observed linewidth broadening most probably originates from the rotational Brownian motion of the nanodiamonds. However, further experimental studies are necessary to fully conclude that the cause of the present observation is the rotational Brownian motion. In particular, the ODMR linewidth broadening must be quantified in relation to the nanodiamond particle size. A promising approach to measure the nanodiamond size and the ODMR linewidth simultaneously is to use the balanced homodyne detection of the probe laser scattering that has recently emerged from studies on quantum optomechanics using levitated nanodiamonds in a vacuum^39–45^. Implementing this technique into a liquid environment may clarify the full mechanism of ODMR linewidth broadening and quantify the present results in relation to the exact nanodiamond size.

Despite of these unresolved issues, the present observation suggests attractive applications of this rotational-motion quantum sensing to various fields like nanofluidics or biological sensing. On the nanoscale, the persistent photostability of NV centers, together with the present rotational-motion sensing, will make nanodiamonds indispensable tools to investigate nanoscale fluid mechanics. The classical experimental tool to visualize nanofluids is organic-molecular fluorescent probes^46,47^ that can also be used for fluorescence depolarization spectroscopy^48^. This method, however, suffers from bleaching of dyes and can only be used for a short period of time and in some specific conditions at specific pH and temperature range. Furthermore, the nanoscale volume restricts the number of fluorescent molecules, thereby shortening the observation time further. In contrast, fluorescent nanodiamonds can provide long-term tracking in various pHs and temperature ranges with excellent fluorescence stability. Recent advancement of fabrication technology has enabled the incorporation of NV centers into nanodiamonds smaller than 5 nm^33,49^. One could insert such ultra-small nanodiamonds into structures with a size of tens of nanometers. It is also possible to access the translational Brownian motion at the same time with measuring the rotational Brownian motion by a wide-field imaging technique. Combining NV-fluorescent nanodiamonds with walking protein motors would be interesting because NV centers provide information on the 3D protein motion (rotation or torsion in addition to the translational motion)^6^. Our findings can provide a new method to measure the rotational motion of single nanoparticles and enable the exploration of nano-scale fluid mechanics.

## Methods

### Sample preparation and perfusion chamber

A commercially available nanodiamond suspension (Microdiamant, MSY 0–0.05, median particle size: 25 nm) was purified five times by a centrifugation of 14,300 RCF for 30 min and the sedimentation was extracted each time in order to remove the sp2-like carbon soot that gives background fluorescence. A small droplet of the suspension was spin-coated on a cleaned coverslip. The spin-coated samples were raster-scanned with an atomic force microscope (Bruker, Edge) to obtain topography images. The peak heights of the distributed nanodiamonds were measured to determine the particle size distributions. A 25 μm-thin copper wire was placed on the coverslip as a microwave linear antenna, and both ends were soldered to SMA connectors. An acrylic spacer with a height of about 4 mm with inlet and outlet tubes was then glued on top of the sample using a UV-curing resin. It was sealed with a glass plate to make a perfusion chamber. The nanodiamonds were detached from the substrate by changing the pH of the buffer solution stepwise in the perfusion chamber. We first sent distilled water to the chamber. A flow of pH-buffer solution (0.1 M sodium carbonate with 5% HCl) was next circulated. During the optical excitation, the continuous flow of these solutions with a rate of 80 μL · min^−1^ was maintained to prevent photothermal accumulation of nanodiamonds.

### Optical measurements

The perfusion chamber was mounted on a 3-axis piezo stage in a home-built confocal fluorescence microscope. A continuous-wave 532 nm laser was used for the excitation with an excitation intensity of 94 kW·cm^−2^ (250 μW). An oil-immersion microscope objective with a numerical aperture of 1.4 was used both for the excitation and the fluorescence collection. The NV fluorescence was filtered by a dichroic beam splitter (Semrock, FF560-FDi01) and a long-pass filter (Semrock, BLP01-561R) to remove the residual green laser scattering. It was then coupled to an optical fiber acting as a pinhole (Thorlabs, 1550HP, core diameter ~10 μm). The fiber-coupled fluorescence was finally guided into a Hanbury-Brown-Twiss (HBT) setup consisting of two APDs (Perkin Elmer SPCM AQRH-14) and a 50:50 beam splitter or connected to a spectrometer equipped with a liquid-nitrogen-cooled CCD camera (Princeton, LNCCD). By scanning the sample with the piezo stage, we were able to obtain the fluorescence scanning images. A time-correlated single-photon counting module (PicoQuant, TimeHarp-260) was used to obtain second-order photon correlation histograms.

### ODMR measurements

Microwave generated from a source (Rohde & Schwarz, SMB100A) was boosted by 45 dB with an amplifier (Mini-circuit, ZHL-16W-43+) and was fed to the microwave linear antenna in the perfusion chamber. The microwave excitation power was 35 dBm (3.2 W) in the impedance mismatching condition. To extract the ODMR spectra from the fluorescence fluctuation of the nanodiamonds, the APD detection was gated for microwave irradiation ON and OFF states by using an RF switch (Mini-circuit, ZYSWA-2-50DR-S) and a bit pattern generator (Spincore, PBESR-PRO-300). The gate width was 200 common for both gates, followed by a laser shut-off time of 100 μs, giving ${I}_{PL}^{ON}$ and ${I}_{PL}^{OFF}$. The repetition rate of the gating pulses was 2 kHz. No external magnetic field was applied.

## Electronic supplementary material

SUPPLEMENTARY INFORMATION

## Data Availability

The datasets generated during and/or analyzed during the current study are available from the corresponding author on reasonable request.
