# Supplementary material for: Observation of the linewidth broadening of single spins in diamond nanoparticles in aqueous fluid and its relation to the rotational Brownian motion
Source: Sci Rep. 2018 Oct 3;8:14773. doi: 10.1038/s41598-018-33041-6 (PMC6170451; doi:10.1038/s41598-018-33041-6)
Supplement: Supplementary file 1 — SUPPLEMENTARY INFORMATION [file 41598_2018_33041_MOESM1_ESM.pdf]

Supplementary material: Observation of the linewidth broadening of single spins in diamond nanoparticles in aqueous fluid and its relation to the rotational Brownian motion

Masazumi Fujiwara,[*,†,‡] Yutaka Shikano,[¶,§,‖,⊥] Ryuta Tsukahara,[‡] Shinichi Shikata,[‡] and Hideki Hashimoto[‡]

†*Department of Chemistry, Osaka City University, 3-3-138, Sugimoto, Sumiyoshi, Osaka 558-8585, Japan*

‡*School of Science and Technology, Kwansei Gakuin University, Sanda, Hyogo, 669-1337, Japan*

¶*Institute for Molecular Science, National Institutes of Natural Sciences, Okazaki, Aichi, 444-8585, Japan*

§*Institute for Quantum Studies, Chapman University, Orange, California 92866, USA*

‖*Research Center for Advanced Science and Technology (RCAST), The University of Tokyo, Meguro-ku, Tokyo, 153-8904, Japan*

⊥*Quantum Computing Center, Keio University, Hiyoshi, Yokohama 223-8522 Japan*

E-mail: masazumi@osaka-cu.ac.jp

1 New spots generated by 532-nm green laser excitation

Figure S1 shows a confocal scanning image of the nanodiamonds in the buffer solution at pH = 9.1. The data are the same as in Fig. 2(b) but have arrows indicating several blurred spots. These spots were created by the green laser excitation when we stopped the liquid flow during the experiment. Without the continuous liquid flow, the green laser excitation gradually generates such fluorescent spots. The fluorescence finally grows brighter than the single NV fluorescence if the liquid flow is stopped for a long period of time. This is probably because nanodiamonds detached from other locations (beyond the imaging region) are accumulated around the laser spot by the strong laser excitation used for the NV detection (optical forces, laser heating, etc). [1] This phenomenon is more prominent in the lower pH buffer solutions (acidic), which may be related to the zeta potential of the nanodiamonds, as nanodiamonds show lower negative zeta-potentials in acidic solutions. [2,3]

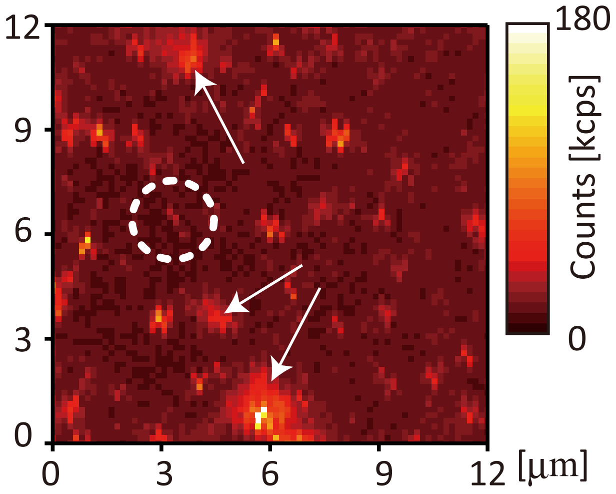

Figure S1: Confocal scanning image of Fig. 2(b) indicating the fluorescent blurred spots that were created by the laser excitation when the liquid flow was stopped a few times. The dashed circle indicates the position of the detached nanodiamond. The arrows indicate the new spots.

2 Experimental setups

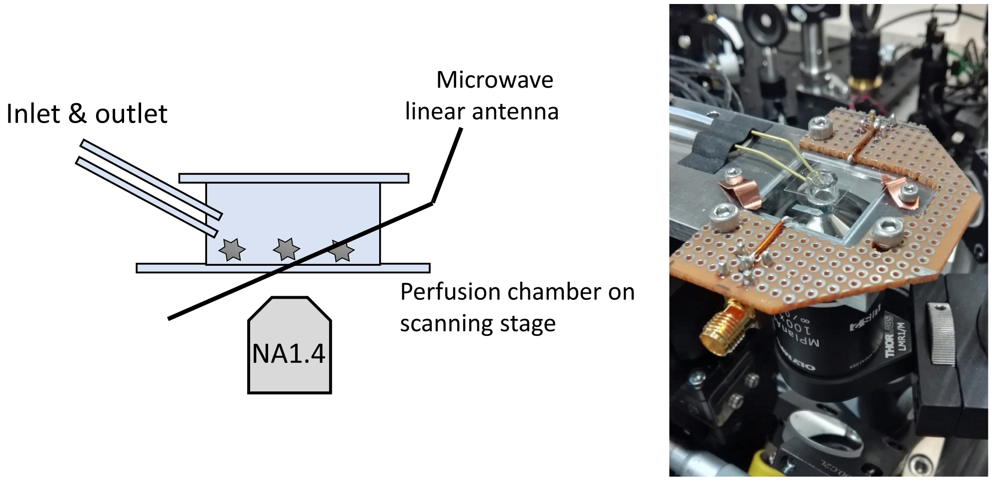

Figure S2: Cross-sectional view and picture of the perfusion chamber.

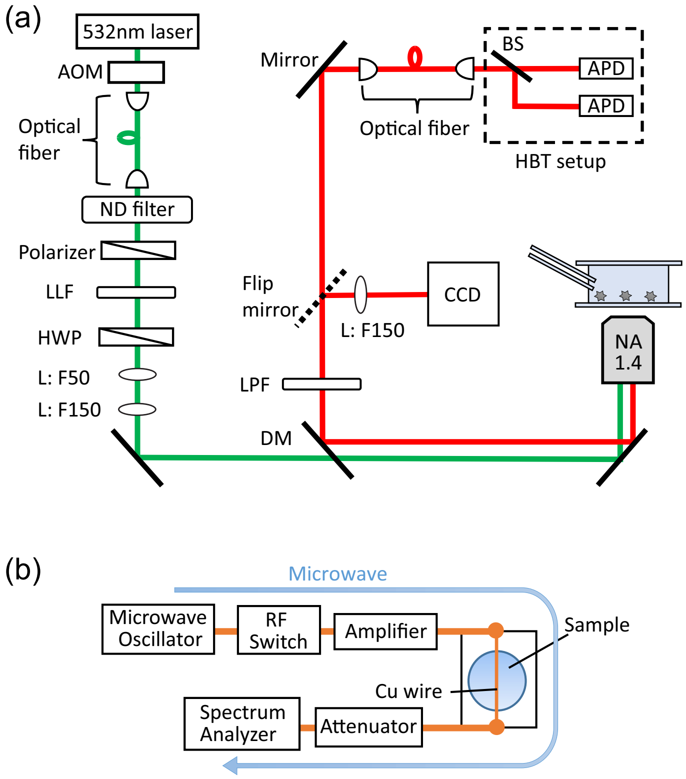

Figure S3: Schematic drawings of the experimental setup for the optical layout (a) and the microwave circuit (b). AOM: acousto-optic modulator. ND: neutral density. LLF: laser line filter. HWP: half-wave plate. L: lens. DM: dichroic mirror. LPF: long-pass filter. CCD: charge coupled device camera. BS: beam splitter. APD: avalanche photodiode.

3 Justification of using single Lorentzian spectral shape

Table S1: Parameters used for reproducing the minor associated peak. We used the following Lorentzian profile: $y_0 + \frac{2A}{\pi}\frac{w}{4(x-x_c)^2+w^2}$, where y_0, A, w, x_c are the offset, peak area, linewidth, and peak position frequency, respectively. $2A/\pi w$ is ODMR contrast.

	Fitting in Fig. 3(b)	Reproduced in Fig. 3(c)
y_0	1.00817	1.00467
A	-2.75×10^{-4}	-1.79×10^{-4}
w	12.09 MHz	13.89 MHz
x_c	2.90359 GHz	2.90519 GHz
$2A/\pi w$	-0.0145	-0.0082

4 The effect of the buffer solutions to the NV spin resonance lines

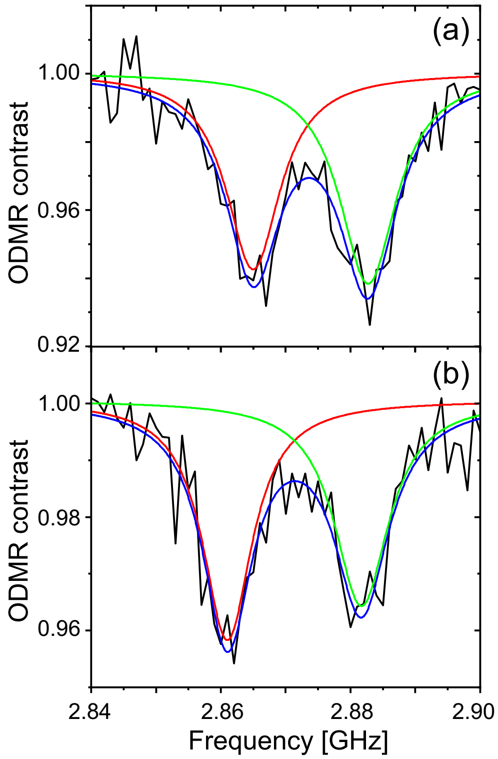

Figure S4: The ODMR spectra of single NV centers in nanodiamonds for different liquid environment. (a) The nanodiamond is immersed in the distilled water and (b) the water is replaced with pH-8.8 buffer solution.

References

(1) Nishimura, Y., Nishida, K., Yamamoto, Y., Ito, S., Tokonami, S., Iida, T. Control of submillimeter phase transition by collective photothermal effect. *J. Phys. Chem. C* **2014**, *118*, 18799–18804.

(2) Williams, O. A., Hees, J., Dieker, C., Jäger, W., Kirste, L., Nebel, C. E. Size-dependent reactivity of diamond nanoparticles. *ACS Nano* **2010**, *4*, 4824–4830.

(3) Ginés, L., Mandal, S., Cheng, C.-L., Sow, M., Williams, O. A. Positive zeta potential of nanodiamonds. *Nanoscale* **2017**, *9*, 12549–12555.
